# Insuffizienzfraktur der Klavikula nach Implantation einer inversen Schulterendoprothese

**DOI:** 10.1007/s00132-021-04205-6

**Published:** 2022-01-06

**Authors:** Laura Elisa Streck, Lothar Seefried, Franca Genest, Thomas Reichel, Maximilian Rudert, Kilian Rueckl

**Affiliations:** Lehrstuhl für Orthopädie der Julius-Maximilians-Universität Würzburg, Orthpädische Klinik König-Ludwig-Haus, Brettreichstr. 11, 97074 Würzburg, Deutschland

**Keywords:** Glenohumeralgelenk, Osteoporose, Osteosynthese, Fraktur, Postoperative Komplikationen, Schulterendoprothetik, Glenohumeral joint, Osteoporosis, Osteosynthesis, fracture, Postoperative complications, Total shoulder replacement

## Abstract

Wir stellen den seltenen Fall einer Insuffizienzfraktur der Klavikula nach Implantation einer inversen Schulterendoprothese (RSA) vor. Als Ursache solcher Frakturen wird eine vermehrte Zugbelastung durch den Musculus deltoideus nach RSA diskutiert. In den wenigen verfügbaren Fallberichten zeigten die betroffenen Patienten deutliche Funktionseinschränkungen. Die Versorgung erfolgte im vorliegenden Fall mit Plattenosteosynthese. Trotz intraoperativ gutem Korrekturergebnis kam es im Verlauf ohne Trauma zum Osteosyntheseversagen mit weiterer Dislokation der Fraktur.

## Abstract (en)

We present a rare case of clavicle fracture following reverse shoulder arthroplasty (RSA). This complication may be caused by the higher tension of the deltoid muscle after RSA, similarly to stress fractures of the acromion. Associated bone disease, i.e. osteoporosis, may contribute. In the few case reports available, the affected patients showed significant functional impairments. In the current case, the fracture was addressed with plate fixation. Despite good intraoperative results, osteosynthesis failure with secondary dislocation of the fracture occurred.

## Einleitung

Die Implantation einer inversen Schulterendoprothese (RSA) ist bei Patienten mit fortgeschrittener Defektarthropathie, Pseudoparalyse und ausgeprägten Glenoiddefekten eine erfolgreiche Therapieoption [3, 13, 14]. Mit zunehmender Anzahl der Primärimplantationen steigt jedoch auch die Zahl der Komplikationen, welche in 6–50 % der Fälle beschrieben werden [2, 14]. Bekannte Probleme sind aseptische Lockerungen, skapuläres Notching, periprothetische Infektionen oder Insuffizienzfrakturen des Akromions [2, 3, 13, 14]. Eine sehr seltene aber nicht weniger relevante Komplikation stellt die Insuffizienzfraktur der Klavikula nach Implantation einer RSA da [1, 8, 9]. Der aktuelle Fallbericht zeigt die Schwierigkeiten der Behandlung dieser Komplikation und diskutiert die verfügbare Literatur.

## Anamnese

**Figure 1:**
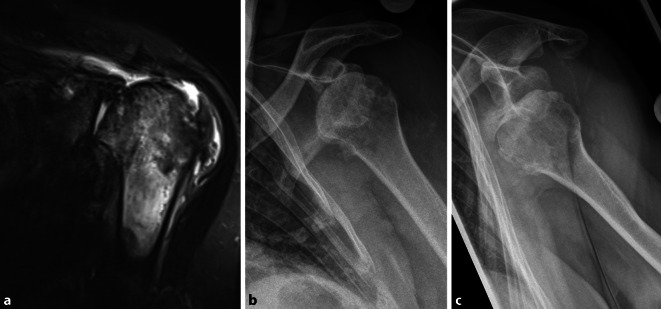

Eine 69-jährige Patientin stellte sich mit progredienten Schmerzen und Bewegungseinschränkung der linken Schulter ohne Ansprechen auf konservative Therapie in unserer Klinik vor. Bei vorbekannter Defektarthropathie (Hamada 4b) offenbarte die konventionelle Röntgenaufnahme eine Schulterluxation (Abb. 1).

Die Patientin hatte multiple Vorerkrankungen. Sie litt an rheumatoider Arthritis und einem fortgeschrittenen Morbus Parkinson (Erstdiagnose 1990) mit rezidivierenden Stürzen. Hinzu kam eine multifaktorielle Osteoporose nach langjähriger Vorbehandlung mit Glukokortikoiden und MTX mit mehreren vorangegangenen, pathologischen Frakturen seit 2005. Ab 2007 erfolgte dahingehend eine medikamentöse Therapie mit wechselnden Präparaten, seit 2016 mit Denosumab. Darüber hinaus bestanden ein knochenmarkzytologisch gesichertes myelodysplastisches Syndrom mit multilinearer Dysplasie mit zytogenetischem (FISH) Deletionsnachweis 7p15 und molekulargenetisch nachgewiesenen BCOR- und DNMT3A-Mutationen sowie klinisch führender Thrombozytopenie. Zudem bestand ein erhöhtes kardiovaskuläres Risiko bei arterieller Hypertonie und Zustand nach tiefer Beinvenenthrombose sowie Lungenembolie. Nach differenzialtherapeutischer Abwägung wurde daher auf eine osteoanabole Intervention mit Teriparatid/Romosozumab verzichtet.

**Figure 2:**
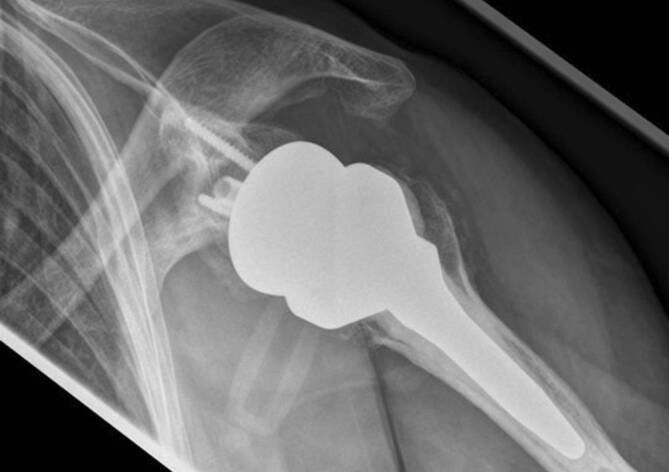

Aufgrund der ausgeprägten Schmerzen und der massiven Einschränkung der Alltagsaktivitäten wurde die Indikation zur Implantation einer RSA gestellt. Es erfolgte die komplikationslose Implantation einer Tornier Aequalis Reversed II Prothese (Wright Medical Group N. V., Memphis, TN, USA) mit zementiertem Schaft. Bei anteriorer Defektsituation des Glenoids (Walch Typ D) erfolgte die Rekonstruktion durch Fixierung des hälftigen Humeruskopfes mit Magnesiumschrauben (Magnezix®, Syntellix AG, Hannover, Deutschland), gefolgt von der Implantation einer Basisplatte mit langem Post. Die postoperative Röntgenbildgebung ist in Abb. 2 dargestellt. Postoperativ erfolgte die Ruhigstellung in der Schulter-Arm-Orthese sowie die passive Mobilisierung für 6 Wochen. Ab der 7. Woche erfolgte zunehmend auch die aktive Mobilisierung, ab der 9. Woche wurde Physiotherapie mit leichten Widerständen begonnen. Zunächst zeigte sich ein regelrechter postoperativer Verlauf, die Patientin war schmerzfrei. 18 Wochen postoperativ traten nach physiotherapeutischer Beübung der Abduktion zunehmende Schmerzen im Bereich der Klavikula auf.

## Befund

Klinisch zeigte sich eine Stufenbildung der lateralen Klavikula mit irregulärer Beweglichkeit und Krepitationen. Es bestanden keine Einschränkungen der Neurologie oder Durchblutung.

## Diagnose

**Figure 3:**
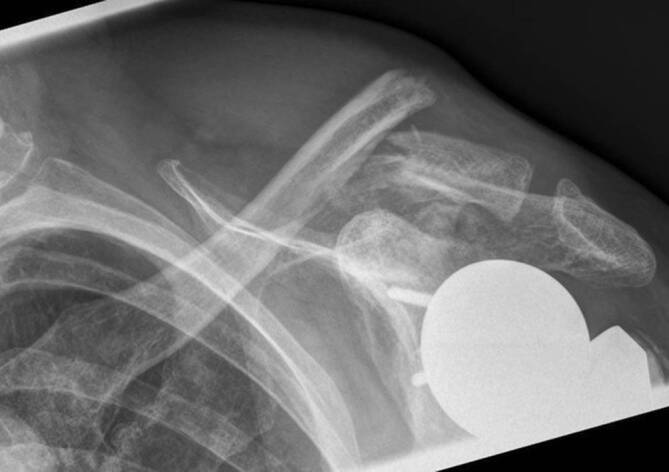

Röntgenologisch bestätigte sich die Verdachtsdiagnose einer lateralen Klavikulafraktur (Robinson 3B1, Jäger Breitner III) mit kranialer Dislokation des medialen Fragments (Abb. 3).

## Therapie und Verlauf

**Figure 4:**
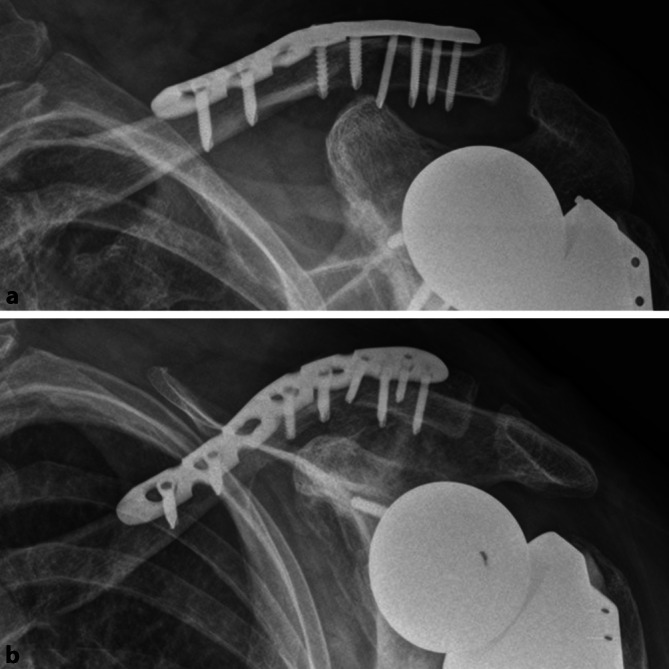

Es erfolgte die komplikationslose Versorgung mittels offener Reposition und winkelstabiler Plattenosteosynthese (5-Loch LCP mit EXT; Synthes Inc., West Chester, PA, USA). Die postoperative Röntgenkontrolle ist in Abb. 4 dargestellt. Die osteologische Medikation wurde unter Berücksichtigung aktueller Laborparameter und Knochendichtemessungen nochmals durch einen Osteologen (DVO) reevaluiert, hierbei wurde keine Indikation zur Änderung des bestehenden Therapieregimes (Vitamin D, Denosumab) gesehen.

**Figure 5:**
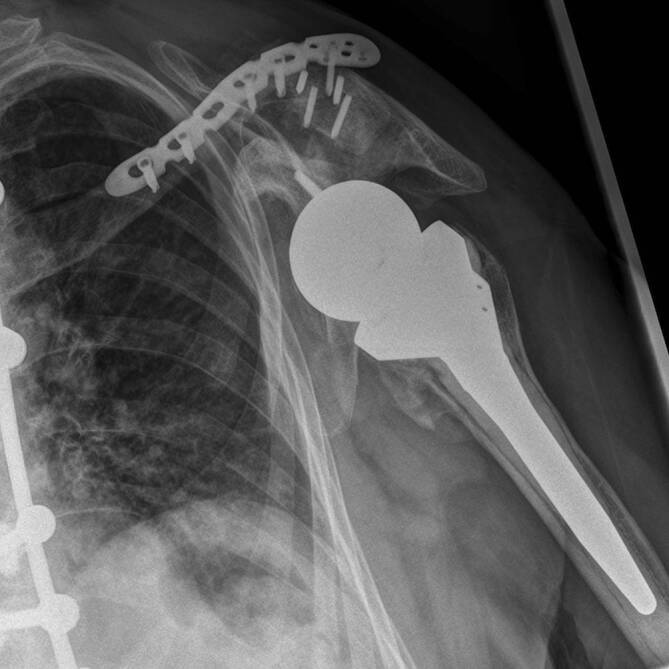

Für die ersten 6 Wochen postoperativ erfolgte die Ruhigstellung in der Schulter-Arm-Orthese mit passiver Mobilisierung und aktiv assistierter Flexion und Abduktion bis 80°. Bei zunächst regelrechtem Verlauf kam es 5 Wochen postoperativ zu vermehrten Schmerzen. Ein auslösendes Trauma wurde von der Patientin glaubhaft verneint. Röntgenologisch zeigte sich die erneute Dislokation des medialen Fragments nach kranial, 4 laterale Schrauben waren abgebrochen, eine laterale Schraube war aus dem Fragment disloziert. Im medialen Fragment bestand röntgenologisch kein Hinweis auf eine Lockerung, die Prothese lag weiterhin regelrecht ein (Abb. 5).

**Figure 6:**
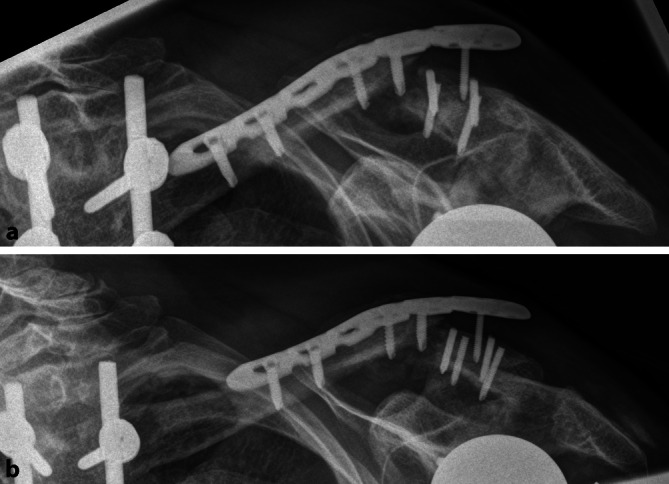

Bei multiplen Vorerkrankungen, limitierter Knochenqualität und Thrombozytopenie (14.000 Thrombozyten/mm^3^) entschieden wir uns gemeinsam mit der Patientin gegen eine Reosyteosynthese mit Hakenplatte und korakoklavikulärer Stabilisierung. In den nachfolgenden Verlaufskontrollen war die Haut- und Weichteilsituation stets unauffällig. Im zeitlichen Verlauf bis 3 Monate postoperativ bestand kein Hinweis auf eine wesentliche sekundäre Dislokation des Osteosynthesematerials oder der Fragmente bei zunehmender Kallusbildung (Abb. 6). Während präoperativ dauerhaft massive Schmerzen (8/10 Punkte nach Numerischer Rating-Skala) bestanden hatten, wurden die Schmerzen nun mit 1/10 nach Numerischer Rating-Skala angegeben. Die Beweglichkeit nach Auftreten der Komplikation war mit Anteversion bis 30° und Abduktion 20° stark eingeschränkt und unverändert zu den präoperativen Befunden. Die Patientin konnte sich jedoch nahezu schmerzfrei auf dem Gehwagen abstützen, sodass die Mobilität der Patientin im Vergleich zur präoperativen Situation deutlich verbessert werden konnte.

Die Patientin wünschte weiterhin keine operative Revision.

## Diskussion

Die RSA wurde seit den ersten Modellen in den 1970er-Jahren konstant weiterentwickelt. Zahlreiche Möglichkeiten zur individuellen Anpassung von Offset, Schaftinklination oder Position der Glenosphäre stehen zur Verfügung. Längst stellt nicht mehr nur die Defektarthropathie eine Indikation zur Implantation einer RSA dar, sondern auch Frakturen mit Ausriss der Tuberkula, Instabilitätsarthrose oder glenoidale Defekte [14]. Insgesamt zeigen sich gute Ergebnisse mit Verbesserung der Schmerzsituation, Beweglichkeit und Funktionalität. Die revisionsfreie Standzeit wird mit 89 % nach 10 Jahren angegeben [5]. Trotz der steten technischen Weiterentwicklung und der insgesamt guten Ergebnisse treten jedoch in bis zu 50 % der Fälle Komplikationen auf. Hierzu zählen insbesondere skapuläres Notching, Lockerungen der Glenoidkomponente, Instabilität der Prothese und periprothetische Infektionen [2].

Akromionfrakturen werden bei bis zu 4 % der Patienten beschrieben [11]. Durch die Verlängerung des Hebelarmes des Musculus deltoideus entsteht eine erhöhte Belastung auf dessen akromialen Ansatz. Insbesondere bei Patienten mit Osteoporose oder rheumatologischer Vorerkrankung kann es zu Insuffizienzfrakturen kommen [10]. Sowohl bei konservativer wie auch operativer Versorgung zeigen sich hohe Pseudarthroseraten. Die langfristigen funktionellen Ergebnisse von Patienten mit Akromionfraktur sind tendenziell schlechter als bei anderen Patienten [11].

Insuffizienzfrakturen der Klavikula stellen dagegen eine Rarität dar. Soweit den Autoren bekannt ist, wurden bislang lediglich 3 Fälle beschrieben [1, 8, 9]. Betroffen waren Patientinnen zwischen 64 und 90 Jahren. Die Frakturen lagen im lateralen Drittel (1 Fall) beziehungsweise am Übergang vom mittleren zum lateralen Drittel der Klavikula (2 Fälle) und traten 6 Wochen bis 10 Monate postoperativ auf. Bei 2 Patientinnen ereignete sich die Fraktur zeitnah nach Aufnahme der aktiven Mobilisierung [1, 8]. Die Versorgung erfolgte in allen Fällen zunächst konservativ mit Ruhigstellung. Bei 2 Patientinnen kam es zur sekundären Dislokation [1, 9]. In beiden Fällen wurde die Indikation zur Plattenosteosynthese gestellt, eine Patientin lehnte die Operation ab [9]. In allen Fällen zeigte sich auch über ein Jahr nach Fraktur noch eine deutliche Funktionseinschränkung [1, 8, 9].

Ätiologisch scheint eine Ermüdungsfraktur durch die erhöhte Zugbelastung des Musculus deltoideus, Pars clavicularis, vergleichbar den Frakturen der Spina scapulae und des Akromions, plausibel. Auch eine vermehrte Belastung der Klavikula bei Kraftübertragung über das Akromioklavikulargelenk ist denkbar, diese besteht insbesondere bei Abduktionsbewegungen. Als weiterer Mechanismus wird zudem eine bereits präoperativ bestehende Vorschädigung der Klavikula diskutiert [1, 8, 9]. Kim et al. sahen die Möglichkeit, dass bei Läsion des Musculus subscapularis durch eine anteriore Migration des Humeruskopfes auch vermehrter Stress auf die Klavikula wirken kann [8]. Eine ausgiebige Literaturrecherche konnte keine Studien finden, welche entsprechende biomechanische Überlegungen für Patienten mit RSA überprüft haben. Im aktuellen Fall bestand präoperativ kein Hinweis auf eine Pathologie der Klavikula.

Die Begleiterkrankungen, konkret der Morbus Parkinson mit erhöhtem Sturzrisiko und die rheumatoide Arthritis, deren erforderliche Therapie sowie das myelodysplastische Syndrom und die konsekutiv langjährig bestehende Osteoporose sollten aus Sicht der Autoren als komplizierende Faktoren beachtet werden. Angesichts der langjährigen antiresorptiven Vorbehandlung in Verbindung mit der für die Knochenregeneration kritischen Glukokortikoid- [7] und MTX- [12] Therapie, erscheint pathophysiologisch eine osteoanabole Therapiesequenz zunächst naheliegend, wenngleich die Datenlage dahingehend inkonsistent ist [6]. Angesichts des Risikos einer Progression des myelodysplastischen Syndroms im Zuge einer forcierten Zellpropagation unter dem Einsatz von Teriparatid sowie des erhöhten Risikos für kardiovaskuläre Endpunkte bei entsprechendem Ausgangsrisiko unter Romosozumab entschieden wir uns gegen einen solchen Behandlungsansatz.

Durch die Implantation einer inversen Prothese wird die Vorspannung des Musculus deltoideus erhöht. Es scheint denkbar, dass hierdurch auch die Zugbelastung am Ursprung des Muskels an der lateralen Klavikula steigt und damit die Biegelast an der medialen Begrenzung des Muskelursprungs. Diese Stelle befindet sich am lateralen Drittel/Übergang vom mittleren zum lateralen Drittel der Klavikula und somit in der Lokalisation, in der die Klavikulafrakturen beschrieben wurden. Aus Sicht der Autoren sind eine sekundäre Dislokation und Pseudarthrosen unter konservativer Therapie somit wahrscheinlich. Wir entschieden uns daher für die Versorgung mit Plattenosteosynthese. Die laterale Extension der winkelstabilen Platte erlaubte eine multidirektionale Verspannung des lateralen Fragmentes und erhöhte hierdurch nochmals den Ausrisswiderstand. Allerdings erfolgte aufgrund der Frakturmorphologie eine bifragmentäre Schraubenpositionierung in der Frakturzone (Abb. 5). Möglicherweise hat die hierdurch fehlende Schwingstrecke der Platte die Schraubenbrüche begünstigt. Retrospektiv wäre eine Osteosynthese nach dem Fixateur-interne-Prinzip oder eine zusätzliche Fixation über den Processus coracoideus mit Fadenzugsystem wahrscheinlich überlegen gewesen. Die oben beschriebenen Faktoren, welche als Ursache der Klavikulafraktur nach RSA diskutiert werden, scheinen auch beim Versagen der Osteosynthese eine Rolle zu spielen. Erschwerend kamen im aktuellen Fall die langjährige Osteoporose und die unwillkürlichen Belastungen der Schulter durch einen erhöhten Muskeltonus sowie einen dauerhaften Tremor der oberen Extremitäten bei Morbus Parkinson hinzu.

Die Insuffizienzfraktur der Klavikula stellt eine sehr seltene und komplexe Komplikation nach RSA-Implantation dar. Unabhängig von konservativer oder operativer Versorgung scheinen die Frakturen auch mittelfristig mit Limitationen der Schulterfunktion einherzugehen. In den in der Literatur beschriebenen Fällen zeigten sich keine zufriedenstellenden Ergebnisse unter konservativer Therapie [1, 8, 9]. Dennoch sollte nach Meinung der Autoren die osteosynthetische Versorgung kritisch diskutiert werden. Insbesondere bei limitierter Knochenqualität und Begleiterkrankungen bestehen begrenzte Erfolgsaussichten mit dem Risiko sekundärer Komplikationen [4].

## Fazit für die Praxis

- Die Klavikulafraktur nach Implantation einer inversen Schulterendoprothese ist eine selten beschriebene Komplikation. In den beschriebenen Fällen zeigten sich nach konservativer wie auch operativer Therapie negative funktionelle Auswirkungen.
- Die konservative Therapie scheint mit hohen Raten sekundärer Dislokationen einherzugehen.
- Die Indikation zur Osteosynthese sollte aus Sicht der Auroren aber insbesondere bei Begleiterkrankungen wie Osteoporose äußerst kritisch abgewogen werden, da die Gefahr eines Versagens der Osteosynthese mit sekundären Komplikationen besteht.
- Erkrankungen wie Osteoporose sollten vor und nach Prothesenimplantation optimal medikamentös eingestellt werden, um das Frakturrisiko zu senken.
